# Characterization of a Type II L-Asparaginase from the Halotolerant *Bacillus subtilis* CH11

**DOI:** 10.3390/life13112145

**Published:** 2023-10-31

**Authors:** Annsy Arredondo-Nuñez, Gisele Monteiro, Carol N. Flores-Fernández, Lina Antenucci, Perttu Permi, Amparo Iris Zavaleta

**Affiliations:** 1 Laboratorio de Biología Molecular, Facultad de Farmacia y Bioquímica, Universidad Nacional Mayor de San Marcos, Lima 01, Peru; annsy.arredondo@unmsm.edu.pe; 2 Department of Pharmaceutical and Biochemical Technology, School of Pharmaceutical Sciences, University of São Paulo, São Paulo 05508-000, Brazil; smgisele@usp.br; 3 Department of Biological and Environmental Science, Nanoscience Center, University of Jyvaskyla, P.O. Box 35, FI-40014 Jyvaskyla, Finland; lina.l.antenucci@jyu.fi (L.A.); perttu.permi@jyu.fi (P.P.); 4 Department of Chemistry, Nanoscience Center, University of Jyvaskyla, P.O. Box 35, FI-40014 Jyvaskyla, Finland; 5 Institute of Biotechnology, Helsinki Institute of Life Science, University of Helsinki, P.O. Box 65, FI-00014 Helsinki, Finland

**Keywords:** L-asparaginase II, *Bacillus subtilis*, saline environment, biochemical characterization

## Abstract

L-asparaginases from bacterial sources have been used in antineoplastic treatments and the food industry. A type II L-asparaginase encoded by the N-truncated gene *ansZP21* of halotolerant *Bacillus subtilis* CH11 isolated from Chilca salterns in Peru was expressed using a heterologous system in *Escherichia coli* BL21 (DE3)pLysS. The recombinant protein was purified using one-step nickel affinity chromatography and exhibited an activity of 234.38 U mg^−1^ and a maximum catalytic activity at pH 9.0 and 60 °C. The enzyme showed a homotetrameric form with an estimated molecular weight of 155 kDa through gel filtration chromatography. The enzyme half-life at 60 °C was 3 h 48 min, and L-asparaginase retained 50% of its initial activity for 24 h at 37 °C. The activity was considerably enhanced by KCl, CaCl_2_, MgCl_2_, mercaptoethanol, and DL-dithiothreitol (*p*-value < 0.01). Moreover, the *V*_max_ and *K*_m_ were 145.2 µmol mL^−1^ min^−1^ and 4.75 mM, respectively. These findings evidence a promising novel type II L-asparaginase for future industrial applications.

## 1. Introduction

L-asparaginase (EC 3.5.1.1) hydrolyzes L-asparagine into aspartic acid and ammonia via an intermediate beta-acyl-enzyme [1,2]. This well-known enzyme is used in cancer therapy, such as childhood acute lymphoblastic leukemia (ALL), non-Hodgkin’s lymphoma, and lymphoid system disorders [3]. The antineoplastic action of L-asparaginase occurs because cancer cells are not able to synthesize enough L-asparagine; depletion of this compound in serum causes the death of cancer cells. However, several side effects have been reported in patients treated with the available L-asparaginases in the market [4,5], such as enzymes from *Escherichia coli* and *Erwinia chrysanthemi*, which can present up to 5% of L-glutaminase activity and clinical resistance of tumor cells during antineoplastic treatment caused by the immune response to the drug from bacterial origin [6,7]. In addition, L-asparaginase has frequently been used in the food industry to reduce the formation of carcinogenic acrylamide, which is generated during heat treatment, and improve taste and nutritional contributions in food [8].

The sources for obtaining this protein are diverse, including plants, animals, bacteria, fungi, and yeasts. Nevertheless, bacterial sources are the most interesting and more comprehensively studied because of their easy handling and genetic manipulation, rapid growth, lower genome complexity, and economically viable production cost [9,10]. Bacteria L-asparaginases are divided into two main classes: type I, which are cytosolic enzymes with low affinity to L-asparagine (*K*_m_ in mM extent); and type II, which are periplasmic enzymes with high affinity to L-asparagine (*K*_m_ in µM limits). The *K*_m_ kinetic parameter is important to direct the industrial enzyme application. Enzymes with high affinity to the substrate are required for human cancer therapy because bloodstream L-asparagine concentrations are in µM range; however, in the food industry, thermostability is the main feature to be considered [11]. 

On the other hand, L-asparaginases from bacteria isolated from hypersaline environments, especially from the *Bacillus* genus, have been described as the most promising anticancer compounds as they show lower immune responses and higher activity [12,13,14]. The genome of *Bacillus subtilis* (*B. subtilis*) has two genes encoding for L-asparaginase (*ansA* and *ansZ)*. The *ansA* gene encodes L-asparaginase I, an intracellular protein with low affinity to the substrate. In contrast, the *ansZ* gene encodes L-asparaginase II, an extracellular enzyme with higher substrate affinity relevant for clinical safety in treatments with L-asparaginase [15,16].

Native L-asparaginases are not highly expressed even by optimizing the growth medium; therefore, the usage of a heterologous expression system represents a great tool for high protein expression, simplicity of purification using specific tags, and ultimately, biochemical, structural, and biophysical studies. Moreover, current studies aim to optimize production media [10,17] and to obtain higher yields of purified protein via different strategies, e.g., including a signal peptide, optimizing a promoter to obtain extracellular proteins [18,19], and truncating the N-terminus of L-asparaginase [15,20]; overall, the target is to obtain better quality, efficiency, and safety of the L-asparaginase.

Studies on type II recombinant proteins from *Bacillus* sp. have been performed to obtain new sources for this pharmaceutically and biotechnologically important protein to meet industrial demands. Thus, several groups have reported the outstanding characteristics of L-asparaginases in terms of thermostability [8], high substrate affinity [2,21], enzymatic activity, and purity [22,23]. However, native and recombinant L-asparaginases might have limitations in their therapeutic effect and intrinsic half-life; therefore, further studies are required in the pharmaceutical setting in combination with nanoparticle systems [24].

This paper describes the cloning, heterologous expression, and purification of N-terminally truncated type II L-asparaginase of *B. subtilis* CH11 from Chilca salterns in Lima, Peru. We have characterized its thermostability in addition to the contribution of temperature, pH, and co-factors to the enzymatic activity. We also determined the kinetic parameters of the enzyme.

## 2. Materials and Methods

### 2.1. Bacteria Strains, Medium, and Chemicals

*Bacillus subtilis* CH11 strain isolated from Chilca Salterns in Lima, Peru, belonged to the collection of the Molecular Biology Laboratory, Faculty of Pharmacy and Biochemistry, Universidad Nacional Mayor de San Marcos. This strain was conserved at −80 °C in TSB medium/ glycerol 30% (*v*/*v*). TSB medium (g/L): casein peptone, 17; K_2_HPO_4_, 2.5; glucose, 2.5; NaCl, 5; soya peptone, 3; pH 7.3. LB-Miller (g/L): yeast extract, 5; peptone from casein, 10; NaCl, 10; pH 7.0. T4 DNA ligase, Phusion DNA polymerase, and *Escherichia coli* BL21(DE3)pLysS were purchased from Thermo Scientific^®^ (Waltham, MA, USA). Restriction endonucleases were obtained from New England Biolabs^®^ (Ipswich, MA, USA). The QIAprep^®^ Spin Miniprep Kit was acquired from QIAGEN (Hilden, Germany). pET-15b and BugBuster^®^ Master Mix were from Novagen^®^ (Merck—Darmstadt, Germany). Finally, the Bicinchoninic Acid Kit and Isopropyl β-D-thiogalactopyranoside were procured from Sigma-Aldrich^®^ (St. Louis, MO, USA).

### 2.2. Bioinformatic Analysis

The native *ansZ* gene sequence was analyzed through the SignalP—6.0 server (DTU Health Tech, Kgs. Lyngby, Denmark) to identify the presence of signal peptide. The molecular weight and isoelectric point were predicted using ProtParam (SIB Bioinformatics Resource, Lausanne, Switzerland). The monomer’s structure was predicted using AlphaFold2 (EMBL-EBI, Cambridge, UK).

### 2.3. Cloning of the ansZP21 Gene Encoding L-ASNasaZP21

The *B. subtilis* CH11 strain was grown in TSB medium for 24 h at 37 °C. The genomic DNA was extracted according to Montes et al. [25]. The *ansZ* gene without the signal peptide YccC (first 60 base pairs), denominated as *ansZP21*, was amplified by PCR from the extracted DNA of *Bacillus* sp. CH11 using the forward primer 5′-TTT **CAT ATG** CCA CAT TCT CC T GAA ACA AAA GAA TCC CC-3′ and the reverse primer 5′-TGC C**GG ATC C**TC AAT ACT CAT TGA AAT AAG C-3′. The gene was cloned using the restriction enzymes *Nde*I and *Bam*HI, whose recognition sequences are in bold in the primers detailed above. PCR was carried out using Phusion DNA polymerase (2 U µL^−1^); the reaction conditions were an initial denaturation at 98 °C for 30 s, followed by 35 cycles at 98 °C for 10 s, 58 °C for 30 s, 72 °C for 20 s, and a final extension at 72 °C for 5 min (T100 Thermal Cycler, Bio-Rad, Hercules, CA, USA). The PCR products were cloned into pET-15b using 1 U of T4 DNA ligase and transformed into *Escherichia coli* DH5α. Then, the plasmids were extracted using kit QIAprep^®^ Spin Miniprep Kit and sent for sequencing to confirm the correct cloning of the *ansZP21* gene. The correct expression vector was transformed into *Escherichia coli* BL21(DE3)pLysS host cells.

### 2.4. Expression and Purification of L-ASNasaZP21

*Escherichia coli* BL21(DE3)pLysS cells were used for protein expression. Cells were grown on 500 mL LB-Miller medium supplemented with 100 µg mL^−1^ ampicillin at 37 °C, 230 rpm on an orbital shaker. The protein expression was induced by adding Isopropyl β-D-thiogalactopyranoside (IPTG) to a final concentration of 0.5 mM when the OD_600_ reached 0.6. Post-induction, the culture was incubated for 14 h at 22 °C and 230 rpm. Subsequently, the cells were harvested by centrifugation at 2133 g for 20 min at 4 °C; the pellet was washed with 1X PBS buffer (pH 7.4) and disrupted using BugBuster^®^ Master Mix reagent, following the manufacturer’s instructions. The clarified lysate containing 6X-His-tagged L-ASNasaZP21 was recovered by centrifugation at 12,555 g for 30 min at 4 °C and used for purification by immobilized metal affinity chromatography (IMAC) using an FPLC system (ÄKTA start, GE Healthcare, Chicago, IL, USA). Briefly, the clarified lysate in 50 mM Tris-HCl containing 100 mM NaCl and 20 mM imidazole, pH 8.5, was loaded onto a pre-equilibrated HisTrap^TM^ FF column of 5 mL (GE Healthcare) at a flow rate of 1 mL min^−1^. Unbound proteins were eliminated by washing the column with a 5 column volume (CV) of the buffer. Finally, the enzyme was eluted by a linear gradient of imidazole (up to 500 mM), desalted in Tris-HCl pH 8.5, and stored at 4 °C for further analysis.

### 2.5. Molecular Weight Determination

The molecular weight of the purified L-ASNasaZP21 was determined by size exclusion chromatography using a HiPrep^TM^ 16/60 Sephacryl^®^ S-200 HR column (GE Healthcare) and 50 mM Tris-HCl containing 100 mM NaCl, pH 8.5, at a flow rate of 0.5 mL min^−1^. The standard curve was derived using a Protein Standard Mix, 15–600 kDa (Sigma-Aldrich^®^, St. Louis, MO, USA), composed of ρ-aminobenzoic acid (0.14 kDa), ribonuclease A type I-A (13.7 kDa), grade VI albumin (44.3 kDa), γ-globulin (150 kDa), and thyroglobulin (670 kDa). The molecular weight was estimated on a semi-log graph following the method described by Mahajan et al. [26].

### 2.6. SDS-PAGE and Zymography

The purity fraction of the L-ASNasaZP21 was evaluated by SDS-PAGE using β-mercaptoethanol as a reducing agent. Zymography was utilized to assess the L-asparaginase activity in situ following electrophoresis with 5% polyacrylamide gel. The gel was incubated in a solution containing 25 mL of 50 mM Tris-HCl pH 8.6, 2 mL of 189 mM L-asparagine, 2 mL of 2 M hydroxylamine, and 1.6 mL of 2 M NaOH. The incubation was performed at 37 °C for 20 min in a Mini Rocker Platform (Bio-Rad). Finally, the gel was stained with a solution containing 10% FeCl_2_, 5% trichloroacetic acid (TCA), and 0.66 M HCl, which enabled visualization of a positive reaction based on the L-aspartic acid β-hydroxamate (AHA) colorimetric assay [27].

### 2.7. L-asparaginase Activity and Protein Assay

The L-asparaginase activity was evaluated using Nessler’s method, with some modifications [28]. The reaction consisted of 100 μL of 50 mM Tris-HCl pH 8.6, 10 μL of 189 mM L-asparagine, 90 μL of H_2_O, and 10 μL of sample. This mixture was incubated for 10 min at 37 °C and stopped using 10 μL of 1.5 M trichloroacetic acid. A volume of 25 μL of the previous reaction was mixed with 25 μL of Nessler solution and 200 μL of H_2_O, and the released free ammonia was quantified, measuring the absorbance at 436 nm. In the negative control, H_2_O was used instead of the enzyme, and the reaction was stopped for the blank before adding the enzyme. A standard calibration curve was derived using different known concentrations of ammonium sulfate between 0.005 and 0.109 µmoles. One unit of enzyme (U) produces 1.0 µmole of ammonia from L-asparagine per minute under optimum conditions.

Protein concentrations were measured according to the Bicinchoninic Acid Kit for 96-well plate-assays, following the manufacturer’s instructions. Bovine serum albumin (BSA) (Sigma-Aldrich^®^, St. Louis, MO, USA) was used as a standard at intervals within 80 to 800 µg of protein.

### 2.8. Biochemical Characterization

The temperature effect on L-ASNasaZP21 activity was investigated between 22 and 80 °C at a fixed pH equal to 8.6. The pH effect was evaluated between 3.0 and 10.0 using appropriate buffers: pH 3.0–5.0, 50 mM sodium citrate, pH 6.0–7.0, 50 mM sodium phosphate, pH 8.0–9.0, 50 mM Tris-HCl buffer, pH 10.0, sodium bicarbonate-NaOH. The temperature was fixed at 60 °C. The results were expressed as the relative activity (%).

The half-life of L-ASNasaZP21 at 22, 37, and 60 °C was determined by incubation from 1 to 24 h, and the residual activity was measured at 60 °C for 10 min via the Nessler method with modifications described above; a control sample without incubation was used. The reaction rate describing heat inactivation was calculated by plotting the time (h) along the x-axis vs. the logarithmic residual activity along the y-axis. The inactivation rate constant (*k*) was estimated using linear regression [29]:

ln [A]_t_ = −*kt* + ln [A]_0_ (1)

where [A]_0_ is the control activity (100%) and the [A]_t_ is the activity at an indicated time, *t* (h). The half-life was determined through the following equation [30]:

*t_(_*_1/2)_ = ln(2)*k*^−1^ (2)

The effect of inhibitors and ions was examined following the same protocol described above and supplementing the standard reaction mixture with appropriate inhibitors and salts. The tested inhibitors were PMSF, Urea, Mercaptoethanol, DL-Dithiothreitol, SDS, and EDTA at final concentrations of 10 mM, and Glutathione at final concentration of 5 mM. The tested salts with mono and divalent cations were NaCl, KCl, CaCl_2_, MgCl_2_, MnCl_2_, BaCl_2_, CuCl_2_, and CoCl_2_, all at final concentrations of 100 mM. The enzyme activity was expressed as the relative activity (%) compared with the control without any supplemented component.

The kinetic assay reactions were carried out at pH 9, 60 °C, and with an incubation time of 10 min. The substrate was tested in a concentration range from 2 to 14 mM. The *V*_max_ and *K*_m_ values were calculated via the Lineweaver–Burk plot.

### 2.9. Data Collection and Analysis

All the analyses were carried out in duplicate and expressed as the mean ± the standard deviation (SD). Data were evaluated using one-way ANOVA and Dunnett’s multiple comparison test using GraphPad Prism version 10.0.2. software (San Diego, CA, USA), with significance defined as *p* < 0.01.

## 3. Results and Discussion

### 3.1. Cloning of the ansZP21 Gene and Sequence Analysis

The lipoprotein signal peptide type II *YccC* reported in *B. subtilis* was found to be in the N-terminal amino sequence of the *ansZ* gene in the present study [8,31]. This contains a conserved Cys20 residue that forms the cleavage site; studies have suggested that the formation of the mature protein is involved in proper folding, where post-translational diacylglycerol modification of the Cys residue is required for signal peptide release [15,32,33]. In addition, Onishi et al. [15] reported that *E. coli* might not process the signal peptide of L-asparaginase from *Bacillus* sp., resulting in incorrect protein folding and a lower purification yield and purity.

**Figure 1:**
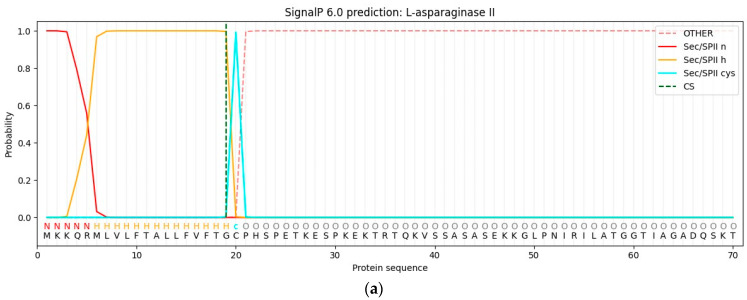

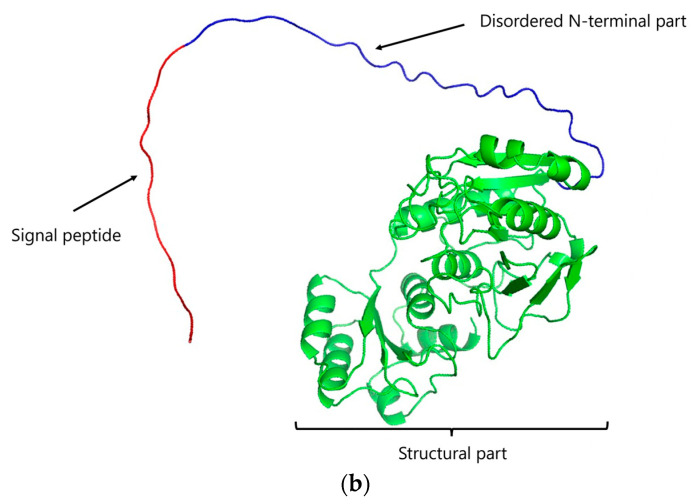
Signal peptide prediction by SignalP—6.0 of *ansZ* gene. (**a**) AlphaFold structure prediction of native *ansZ* gene. (**b**) Signal peptide, disordered N-terminal, and structural parts are shown in red, blue, and green, respectively.

The native *ansZ* encoded protein included a signal peptide identified via bioinformatics analysis on the SignalP—6.0 server with a probability of 0.996% (Figure 1a). This finding is in line with the AlphaFold2 structure prediction of L-asparaginase II from *B. subtilis* (Figure 1b) [34,35]. Therefore, signal peptides between 1 and 19 amino acid residues and Cys20 residue were removed when cloning the protein for *E. coli* expression. Based on that, the *ansZP21* gene has 1068 bps, encoding the protein L-ASNasaZP21 of 355 amino acids, with a molecular weight and isoelectric point of 37.91 kDa and 6.16, respectively.

### 3.2. Expression and Purification of L-ASNasaZP21

*B. subtilis* L-ASNasaZP21 expressed in heterologous *E. coli* BL21(DE3)pLysS and purified showed a specific activity of 234.38 U mg^−1^, which is higher than reported values in similar proteins [15,36,37]. This may result from the better protein solubility and reduced misfolding associated with N-terminal truncation and the optimized expression protocol [20,38]. In addition, Moura et al. [20] reported that *E. coli* BL21(DE3)pLysS (89.0 ± 4.4) expresses L-asparaginase with a higher enzymatic activity compared with other *E. coli* strains (T7 Express Crystal, 57.0 ± 1.7; Tuner (DE3), 41.6 ± 2.0; C43 (DE3), 22.4 ± 1.6; BL21 (DE3), 12.5 ± 1.2; Lemo21 (DE3), 10.9 ± 1.2; SHuffle T7, 4.9 ± 1.9; GroEL (DE3), 2.2 ± 2.1). Thus, it is presumed that *E. coli* strains Tuner (DE3), C43 (DE3), Lem21 (DE3), and (DE3) are not efficient hosts for adequate protein folding. Furthermore, SHuffle T7 does not favor the disulfide bond between Cys99 and Cys127 bonds for the correct structural conformation, and GroEL presents chaperones with no activity at low post-induction temperatures [20].

A purification factor of 85.2-fold and a recovery yield of 61.9% were achieved after the affinity chromatography (Table 1). The N-truncated version of our L-asparaginase was expressed, including an N-terminal 6X-His-tag, which allowed high selectivity to obtain a highly purified protein from a complex sample [8,39]. Studies on other type II L-asparaginases from *Bacillus* sp. using affinity chromatography have been reported, achieving activities of 4438.6 U mg^−1^ [22], 1146 U mg^−1^ [40], and 162.9 U mg^−1^ with a recovery yield of 67.21% [18] (Table 2). Specific activity (U mg^−1^) is clinically relevant because the patient will receive an L-asparaginase dose based on units per body surface area. In clinical terms, this means that a much higher amount of protein (in mg) is necessary to reach therapeutic efficacy for enzymes with low specific activity. In this context, L-ASNasaZP21 has specific activity very similar to that applied in clinical practice [11].

### 3.3. Molecular Weight Determination, SDS-PAGE, and Zymography

**Figure 2:**
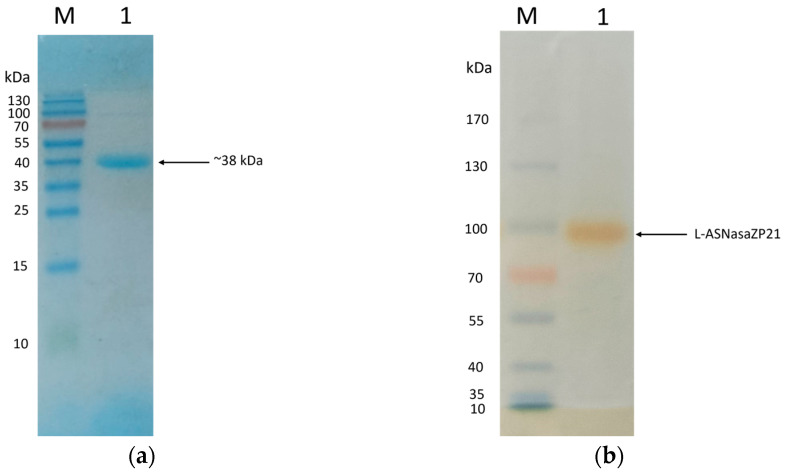
L-ASNasaZP21 analysis by SDS-PAGE (**a**) and zymography (**b**). Lanes: M, PageRuler^TM^ Prestained (Thermo Scientific^®^, Waltham, MA, USA); 1, purified L-ASNasaZP21.

SDS-page analysis showed that the molecular weight of L-ASNasaZP21 was 38 kDa, as expected from the bioinformatic analysis, and the purity grade was relatively high (Figure 2a). The zymography demonstrated the L-asparaginase activity in situ, although the molecular weight did not match that observed in denaturing conditions (Figure 2b). This is presumably associated with the oligomerization of the protein in native conditions. In accordance with this, the molecular weight determined by gel filtration chromatography was 155 kDa, indicating the possible tetrameric structure of L-ASNasaZP21, in agreement with preliminary studies [42,43].

### 3.4. Effect of Temperature and pH

**Figure 3:**
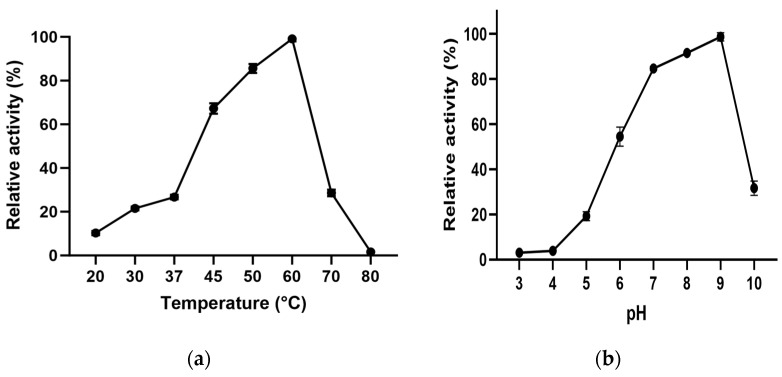
Effect of temperature (**a**) and pH (**b**) on the enzymatic activity of L-ASNasaZP21. The relative activity was expressed as a percentage of the maximum activity. Error bars represent one standard deviation from the mean (*n* = 2).

L-ASNasaZP21 exhibited optimum activity at 60 °C (Figure 3a), 2.7-fold higher than at 37 °C. The enzyme retained more than 60% of its activity at 45 °C and around 30% at 70 °C. The optimum pH of L-ASNasaZP21 was 9.0 (Figure 3b), retaining more than 80% of its activity at physiological pH (pH 7). These results agree with those of Feng et al. [36], who reported an N-truncated L-asparaginase with an optimum temperature of 65 °C. However, this differed from that reported for other type II L-asparaginases from *B. subtilis*, which exhibited optimum activity at 40 °C and pH 7.5 [37], as well as at 37 °C and pH 5.0 [41]. These differences might be because the protein was from a halotolerant bacterium, in line with Lakshmi et al. [44]. Nevertheless, most bacterial L-asparaginases have shown optimum activity between 30 and 50 °C [45] and at pH between 7.0 and 9.0 [46].

### 3.5. Molecular Weight Determination, SDS-PAGE, and Zymography

SDS-page analysis showed that the molecular weight of L-ASNasaZP21 was 38 kDa, as expected from the bioinformatic analysis, and the purity grade was relatively high (Figure 2a). The zymography exhibited the L-asparaginase activity in situ, although the molecular weight did not match that observed in denaturing conditions (Figure 2b). This is presumably associated with oligomerization of the protein in native conditions. In accordance with this, the molecular weight determined by gel filtration chromatography was 155 kDa, indicating the possible tetrameric structure of L-ASNasaZP21, in agreement with preliminary studies [42,43].

### 3.6. Effect of Temperature and pH

L-ASNasaZP21 exhibited an optimum activity at 60 °C (Figure 3a), 2.7-fold higher than at 37 °C. The enzyme retained more than 60% of its activity at 45 °C and around 30% at 70 °C. The optimum pH of L-ASNasaZP21 was 9.0 (Figure 3b), retaining more than 80% of its activity at physiological pH (pH 7). These results agree with those of Feng et al. [36], who reported an N-truncated L-asparaginase with an optimum temperature of 65 °C. However, these findings differed from those reported for other type II L-asparaginases from *B. subtilis*, which exhibited optimum activity at 40 °C and pH 7.5 [37], as well as at 37 °C and pH 5.0 [41]. These differences might be because the protein was from a halotolerant bacterium, in line with Lakshmi et al. [44]. Nevertheless, most bacterial L-asparaginases have shown optimum activity between 30 and 50 °C [45] and at pH between 7.0 and 9.0 [46].

### 3.7. Effect of Metal Ions and Inhibitors

The effects on enzymatic activity of inhibitors and ions are described in Table 3. The activity was slightly enhanced by KCl (1.2-fold) and MgCl_2_ (1.5-fold), while the highest improvement in activity was observed in the presence of CaCl_2_ (3.1-fold). This positive effect of ions on the activity has also been described for L-asparaginases from *B. sonorensis* [22] and *B. amyloliquefaciens* MKSE [8]. On the other hand, some authors have reported the inhibitory effect of MnCl_2_, CuCl_2_, and CoCl_2_ on L-asparaginase activity [36,41].

Likewise, L-ASNasaZP21 activity was enhanced in the presence of Mercaptoethanol (1.4-fold) and DL-dithiothreitol (2.7-fold). Reducing agents might decrease protein aggregation due to intermolecular disulfide bridge formation. These findings are similar to those with L-asparaginases from *Pectobacterium carotovorum* [47] and *Erwinia carotovora* [48]. The activity was partially inhibited by EDTA, while the presence of SDS dropped the activity to zero, as mentioned by other authors [26,49].

### 3.8. Thermostability of L-ASNasaZP21

**Figure 4:**
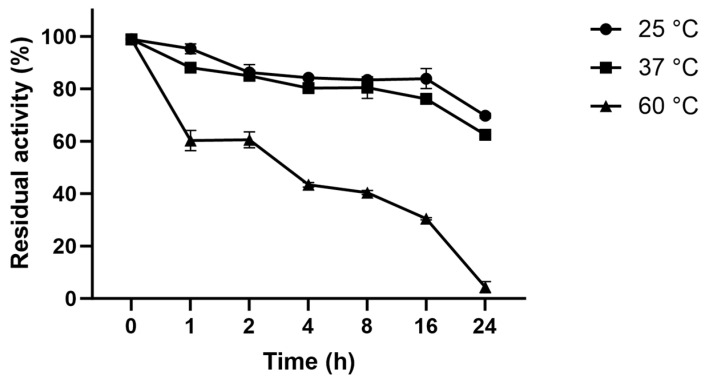
Thermostability of L-ASNasaZP21 at 25, 37, 60 °C. Error bars represent one standard deviation from the mean (*n* = 2).

Figure 4 shows the inactivation process at 22, 37, and 60 °C. The half-life of L-ASNasaZP21 at 60 °C was 3 h 48 min, and it retained around 60% of its activity after 1 h of incubation. At 25 and 37 °C, the half-life was >24 h and retained 50% of its activity after 24 h of incubation. L-ASNasaZP21 showed better thermal stability than previously reported [29,37], which could be promising for industrial applications.

### 3.9. Determination of Kinetic Parameters

**Figure 5:**
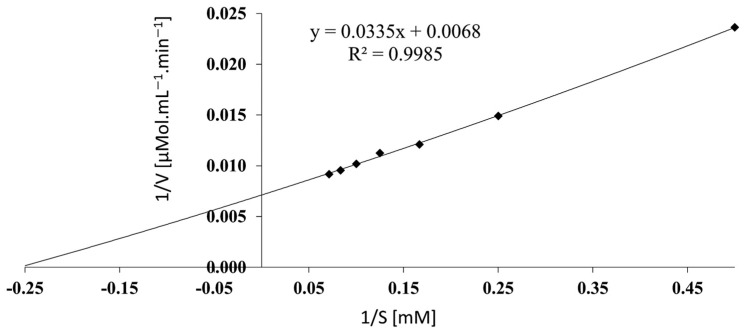
Lineweaver–Burk plot to determine kinetic parameters. L-asparagine concentrations in the x-axis vs. the reciprocal of V_0_ in the y-axis.

The kinetic constants were estimated from the Lineweaver–Burk plot (Figure 5). *V*_max_ and *K*_m_ were 145.2 µmol mL^−1^ min^−1^ and 4.75 mM, respectively. The *K*_m_ value was comparable to the 5.29 mM described by Feng et al. [36] and the 7.06 mM finding from Onishi et al. [15], but clearly in contrast to the 0.43 mM reported by Jia et al. [37]. However, it presents a lower substrate affinity than *Escherichia coli* and *Erwinia chrysanthemi*, with *K*_m_ of 0.02–0.05 and 0.05 mM, respectively [50]. Kinetic parameters are very important to industrial applications; however, in vitro analysis can only partially show the potential of the enzymes due to the simplicity of the systems. For instance, yeast L-asparaginase ScASNase1 doubled its activity when incubated with human serum [51]. Additionally, L-asparaginase from *Erwinia chrysanthemi* changes its kinetic parameter in the presence of osmolytes, increasing its *V*_max_ [52]. Although *Escherichia coli* and *Erwinia chrysanthemi* present similar substrate affinity, the in vivo enzyme stability has led physicians to choose enzymes from *E. coli* as the first-line option in cancer treatment [11]. Thus, the additional characterization of in vitro cancer cytotoxicity, immunogenicity, and in vivo efficacy studies are necessary to evaluate the clinical application of L-ASNasaZP21.

## 4. Conclusions

This study contributes to the existing knowledge of L-asparaginases because it describes a type II protein from *B. subtilis* isolated from a natural extreme environment and reports the biochemical properties of the engineered L-ASNasaZP21. The major modification to the native enzyme sequence included the removal of the signal peptide in the N-terminus; this improved the protein yield in a heterologous expression system, facilitating the purification procedure. The data show that the activity is well retained even if the protein is incubated for a period longer than 3 h at 60 °C; additionally, optimal pH and temperature are slightly higher. These characteristics are particularly interesting for the usage of L-ASNasaZP21 in the pharmaceutical and food industries. Moreover, this study reports a possible multimeric structure of the protein after cleavage of the signal peptide that might be crucial for the catalytic activity; therefore, L-ASNasaZP21 represents an optimal protein construct for improving protein expression and purification and preserving enzymatic activity. Notably, the presence of CaCl_2_ resulted in a 3.1-fold enhancement of the enzyme activity. This is, once more, a valuable characteristic for potential industrial applications.

Subsequent studies, e.g., structural elucidation by experimental methods, will contribute to knowledge of the mechanism of action, dynamics, and interaction with other biomolecules, enabling the further evaluation of L-ASNasaZP21’s potential in food and pharmaceutical industry applications. Additionally, for its pharmaceutical applications, the development of suitable administration systems is required that improve the stability and physicochemical properties of L-ASNasaZP21, while retaining its enzymatic activity in complex with polymers, liposomes, and nanoparticles.

## Figures and Tables

**Table 1:** Summary of the purification of L-ASNasaZP21.

	Total Activity (U)	Total Protein (mg)	Activity (U mg^−1^)	Purification-Fold	Yield (%)
Crude extract	2.38 × 10^2^	86.39	2.75	1.0	100.0
Ni-affinity	1.47 × 10^2^	0.63	234.38	85.2	61.9

**Table 2:** Physicochemical and kinetic characteristics of L-asparaginases of genus *Bacillus*.

Source	pH	Temperature (°C)	Activity (U mg^−1^)	*K*_m_ (mM)	Chromatography ^1^	References
*Bacillus subtilis* CH11	9.0	60	234.38	4.75	AC	This study
*B. subtilis* BDRD-ST26	-	65	162.90	5.29	HIC, IEX, GC	Feng et al. [36]
*B. subtilis* 168	8.0 7.5	65 50	45.40 31.90	2.06 7.06	IEX, HIC	Onishi et al. [15]
*B. subtilis* B11-06	7.5	40	92.45	0.43	HIC	Jia et al. [37]
*B. subtilis* KDPS1	5.0	37	-	-	IEX	Sanghvi et al. [41]
*B. aryabhattai* ITBHU02	8.5	40	680.50	0.25	HIC, GF	Singh et al. [21]
*B. amyloliquefaciens* MKSE	8.5	65	136.30	1.15	AC	Yim et al. [8]
*B. cereus*	9.0	50	550.80	9.38	HIC, IEX, GF	Feng et al. [36]
*B. megaterium* H-1	8.0	40	1146.29	21.63	AC	Lu et al. [40]
*B. sonorensis*	7.0	45	4438.62	2.00	AC	Aly et al. [22]
*B. velenzensis*	7.5	37	31.77	0.04	GF	Mostafa et al. [2]

-, not determined. ^1^ AC (affinity), IEX (ion exchange), HIC (hydrophobic interaction), GC (gel filtration).

**Table 3:** Effect of metal ions and inhibitors on L-ASNaseZP21 activity.

Ions/Inhibitors	Final Concentration	Relative Activity (%) ^1^
Control	-	100.00
KCl	100 mM	124.500 ± 1.85 *
NaCl		106.300 ± 0.09
MgCl_2_		149.800 ± 4.04 *
CaCl_2_		310.700 ± 3.28 *
BaCl_2_		95.070 ± 2.73
MnCl_2_		0.0 *
CuCl_2_		0.0 *
CoCl_2_		0.0 *
PMFS	10 mM	118.700 ± 5.77
Urea		96.260 ± 7.36
Mercaptoethanol		139.800 ± 3.52 *
DL-dithiothreitol		271.100 ± 37.00 *
SDS		0.0 *
EDTA		58.850 ± 1.46 *
Glutathione	5 mM	97.860 ± 4.39

^1^ The relative activity was expressed as the percentage of activity compared with a control without metal ions. Error bars represent one standard deviation from the mean (*n* = 2). * *p* < 0.01 vs. control (ANOVA test).

## Data Availability

Not applicable.
